# Theoretical Treatment of Limitations Inherent in Simple 3D Stimuli: Triangles and the P3P Problem

**DOI:** 10.3390/vision5010010

**Published:** 2021-02-17

**Authors:** Vasiliy Minkov, Tadamasa Sawada

**Affiliations:** School of Psychology, HSE University, 101000 Moscow, Russia; proveyourselfmail@gmail.com

**Keywords:** 3D perception, depth perception, P3P problem, shape constancy, shape ambiguity, visual space

## Abstract

Understanding the visual stimulus in a psychophysical experiment, theoretically, is critical for controlling the experiment, for interpreting the empirical results of the experiment, and for discussing the mechanisms the visual system used to get these results. This fact encourages visual scientists to use “simple” visual stimuli in their experiments. A triangle is one of the simplest stimuli that has been used by psychophysicists to study 3D perception. It has also been used to compose the polygonal meshes that represent complex 3D surfaces in computer graphics. The relationship between the shape, orientation, and retinal image of a triangle has also been studied as the Perspective-3-Point problem (P3P). In this study, the statistical properties of this relationship between the 2D retinal image of a triangle and its recovered 3D orientation were tested in a simulation experiment whose results showed that a triangle is qualitatively different from more complex shapes that have been used to recover 3D information from their retinal images. This raises an important question, namely, how many, if any, inferences about our visual system can be generalized to our perceptions in everyday life when they are based on psychophysical experiments that used very simple visual stimuli such as triangles.

## 1. Introduction

Understanding the visual stimulus in a psychophysical experiment, theoretically, is critical for controlling the experiment, for interpreting the empirical results of the experiment, and for discussing the mechanisms the visual system uses to get these results. This goal has encouraged visual scientists to use “simple” visual stimuli in their experiments. A 2D retinal image can, theoretically, be decomposed into points, contours, gratings, and Gabor patterns. The perception of such stimuli has also been studied in psychophysical experiments (e.g., [1,2]). Our theoretical understanding of these simple elements taken with the empirical studies of their perception permits us to discuss, systematically, the perception of more complex stimuli that are composed of such elements.

The perception of 3D information cannot be studied in the same way that the perception of 2D information on the frontoparallel plane can be studied because projecting a 3D scene on to a 2D retina is a *well-posed forward* problem but recovering this 3D information in the scene from a 2D retinal image is *an ill-posed inverse* problem [3,4]. There are infinitely many possible 3D interpretations of the 2D retinal image. The visual system can resolve this problem by using a priori constraints in the 3D scene, such as mirror-symmetry and volume [4,5,6,7]. The visual system can also use a number of depth cues for perceiving the 3D information [8]. Consider, for example, that human beings normally view a 3D scene with a pair of eyes. These eyes are separated about 6.5 cm so their retinal images of the scene will be slightly different from one another. This difference between the stereo-retinal images is called “binocular disparity”. The visual system can use this cue to perceive depth.

Can a 3D scene or a 3D object be decomposed into its simple parts in the same way that its 2D retinal image has been decomposed? The visual system could segment objects in the scene and process them individually [9,10,11]. These individual objects can even be segmented into smaller components, making it possible to recognize them [12,13,14]. The relationship between these components and their 2D retinal images can be characterized by what has been called their “non-accidental properties” [6,14,15]. These non-accidental properties are invariant features in the images of the components, and these image features can be assumed to play an important role for perceiving 3D information from the 2D retinal image.

Often, there is even more reduction of the visual stimuli employed in computer vision and in computer graphics where 3D scenes and 3D objects are often represented as compositions made up of points, or of polygons. These polygons are used to compose a polygonal mesh that represents, approximately, any complex surface of a scene and of an object. Triangles are commonly used to compose the polygonal mesh because a triangle is the *simplest* polygon that can enclose a surface that is always planar. Note that these triangles and dots are two of the simplest stimuli that have been used to study 3D perception. It has been shown that the 3D perception of these triangles, and dots, as well as some other very simple visual stimuli is *not* veridical while the perception of 3D scenes and objects “out there” is veridical in our everyday life (see References [3,4] for discussions). This difference in the veridicality of the 3D perception can be attributed to the geometrical properties of the stimuli employed. The visual system uses a priori constraints and depth cues to establish veridical 3D perception. Theoretically, both the constraints and the cues require visual stimuli that have at least some geometrical complexity. So, an understanding of the geometrical properties of the stimuli employed is necessary if one wants to understand the difference observed between veridical and non-veridical 3D perception (see References [16,17,18] for a discussion about the importance of Theories in Science).

In this study, we analyzed numerically: (i) the geometrical properties of the relationship between a triangle in a 3D scene and its 2D retinal image, and (ii) the retinal images of triangles that were used as visual stimuli in two prior psychophysical studies. These triangles were discussed in detail in this study because these triangles can be regarded as the kind of elements that can be used to compose a 3D scene. They are the simplest polygons that can enclose planar surfaces, and they can even represent, approximately, a more complex surface by using them to compose a polygonal mesh.

## 2. Analysis

**Figure 1:**
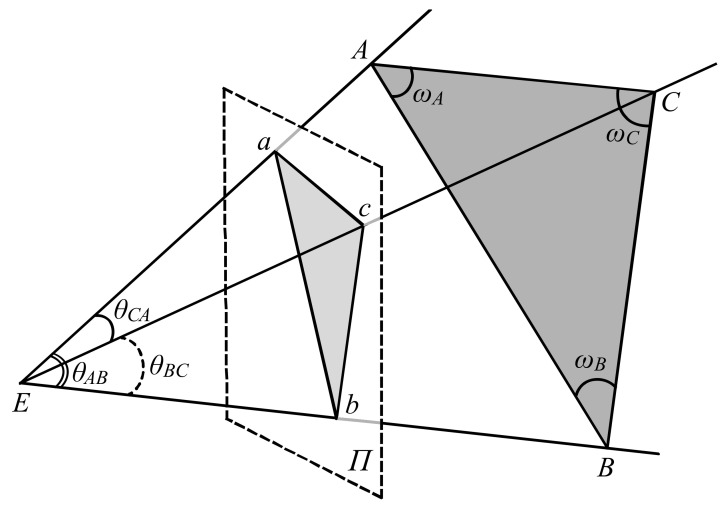
A perspective projection from the triangle *ABC* in a 3D scene to the triangle *abc* in the 2D image plane *Π* from the center of projection *E*. This projection can be represented as the tetrahedron *EABC*.

The relationship between a triangle *ABC* in a 3D scene and its 2D perspective projection to a retina can be represented by the tetrahedron *EABC* shown in Figure 1. The bottom face of the tetrahedron is the triangle *ABC* and the apex *E* represents the center of projection in an eye. The retinal image of *ABC* can be represented by three visual angles *θ_BC_*, *θ_CA_*, and *θ_AB_* at *E*. The shape of the triangle *ABC* can be characterized by two angles *ω_A_* and *ω_B_* at the vertices *A* and *B*. The third angle *ω_C_* of the triangle *ABC* is *ω_C_* = 180°—*ω_A_*—*ω_B_*. The size of *ABC* can be controlled by the length of the line-segment *AB*. The length of *AB* (‖*AB*‖) can be set to 1 without any loss of generality. If the size of *ABC* changes by a factor of *s* (‖*AB*‖ = *s*), the size of the tetrahedron *EABC* changes by a factor of *s* while all of the angles of *EABC* remain constant. Note that the distance and size of *ABC* from the center of projection *E* changes by a factor of *s* while the orientation of *ABC* is unchanged.

First, consider recovering the shape of the triangle *ABC* from its retinal image when both the orientation of *ABC* in a 3D scene and the retinal image are given. The shape can be uniquely determined by finding the intersection of the lines of projection with a plane that has the given orientation. The orientation can be arbitrary unless the normal to the plane is perpendicular to any of the lines of the projection. The distance of the plane from the center of projection *E* characterizes the size of *ABC*.

Next, consider recovering the orientation of the triangle *ABC* from its retinal image when the shape of *ABC* and the retinal image are given. This problem is relevant with, for example, shape recognition, shape reconstruction, and mental rotation tasks. It is known as the Perspective-3-Point (P3P) problem [19,20,21,22,23,24,25,26,27,28]. It has been proven that there are 0, or up to 4, possible 3D interpretations of the triangle for the given shape (*ω_A_*, *ω_B_*, *ω_C_*) as well as for the size *s* and for the retinal image of the triangle given (*θ_BC_*, *θ_CA_*, *θ_AB_*). These interpretations correspond with the solutions of the quartic equation that is used to solve the P3P problem (see Reference [28]).

The P3P problem in our study was formulated as the relationship between the triangle *ABC* and the visual angles *θ_BC_*, *θ_CA_*, and *θ_AB_* [20,21,22,23,24,25]. This allows us to control the retinal image of the triangle *ABC* with only 3 parameters (*θ_BC_*, *θ_CA_*, *θ_AB_*). The recovered orientation of the triangle *ABC* is characterized by the distance *l_A_*, *l_B_*, and *l_C_* of the vertices *A*, *B*, and *C* from the center of projection *E*. The recovered positions of *A*, *B*, and *C* can be written as:

(1) $$\left\{\begin{array}{c} A=l_{A}V_{A}\\ B=l_{B}V_{B}\\ C=l_{C}V_{C} \end{array}\right.$$

where *V_A_*, *V_B_*, and *V_C_* are unit vectors representing the lines of sight from *E* to *A*, *B*, and *C*. The vectors *V_A_*, *V_B_*, and *V_C_* can be derived from the retinal image of the triangle (*θ_BC_*, *θ_CA_*, *θ_AB_*). The distance *l_A_*, *l_B_*, and *l_C_* are restricted to be positive so that the recovered positions of *A*, *B*, and *C* do not extend behind the center of projection *E*.

Note that the P3P problem in computer vision [19,27,28] is formulated as a relationship between *ABC* and its planar perspective image (the triangle *abc* on the image plane *Π* in Figure 1) in a calibrated camera. The visual angles *θ_BC_*, *θ_CA_*, and *θ_AB_* can be computed from this calibrated image.

### 2.1. Monte-Carlo Simulation

We tested the frequencies of the number of possible 3D interpretations of the triangle *ABC* for the retinal image in two Monte-Carlo simulation experiments by using an algorithm developed by Fischler & Bolles [21] for solving the P3P problem. The shapes of the triangle (*ω_A_*, *ω_B_*, *ω_C_*) and the retinal image (*θ_BC_*, *θ_CA_*, *θ_AB_*) were randomly generated in each trial by randomly sampling *ω_A_*, *ω_B_*, *θ_BC_*, *θ_CA_*, and *θ_AB_* from uniform distributions. The sampled variables of *ω_A_*, *ω_B_*, *θ_BC_*, *θ_CA_*, and *θ_AB_* were independent from one another but were constrained so that *ω_A_*, *ω_B_*, and *ω_C_* form the triangle and *θ_BC_*, *θ_CA_*, and *θ_AB_* form an apex of the tetrahedron: *ω_A_* + *ω_B_* + *ω_C_* = 180°, *θ_BC_* + *θ_CA_* + *θ_AB_* < 360°, *θ_BC_* + *θ_CA_* > *θ_AB_*, *θ_CA_* + *θ_AB_* > *θ_BC_*, *θ_AB_* + *θ_BC_* > *θ_CA_*. Additionally, the shape of the triangle was restricted by an additional constraint, namely, 10° < *ω_A_*, *ω_B_*, *ω_C_* < 170°. With this done, the possible 3D interpretations of the triangle for the retinal images (*θ_BC_*, *θ_CA_*, *θ_AB_*) are computed by using an algorithm developed by Fischler & Bolles [21] for solving the P3P problem. This algorithm was implemented in a C++ program. We confirmed that this program is both more reliable and faster than other existing programs that have been used for the P3P problem (Appendix A).

In the first experiment, the ranges of the sampling of *θ_BC_*, *θ_CA_*, and *θ_AB_* were set to 0.1° < *θ_BC_*, *θ_CA_*, *θ_AB_* < *θ*_max_, where *θ*_max_ is an independent variable (2°, 4°, … 118°, 120°). There were 4 × 10^8^ trials for each value of *θ*_max_. In the second experiment, the ranges of sampling were set to *θ*_max_/2 < *θ_BC_*, *θ_CA_*, *θ_AB_* < *θ*_max_.

**Figure 2:**
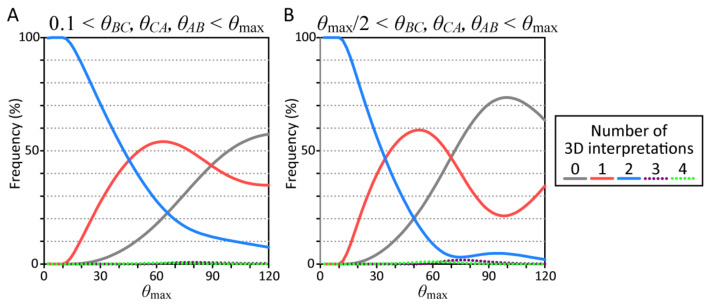
Results of two Monte-Carlo simulation experiments. The ordinate shows the frequency of the numbers of possible 3D interpretations and the abscissa shows *θ*_max_. The five curves show the numbers of possible 3D interpretations. (**A**) The visual angles *θ_BC_*, *θ_CA_*, and *θ_AB_* were sampled between 0.1° and *θ*_max_. (**B**) The visual angles *θ_BC_*, *θ_CA_*, and *θ_AB_* were sampled between *θ*_max_/2 and *θ*_max_.

The results of this simulation are shown in Figure 2. The ordinates show the frequencies of the numbers of possible 3D interpretations. The abscissa shows *θ*_max_ that controls the range of the sampling. The four curves show the numbers of possible 3D interpretations.

These results show the frequency of obtaining two possible interpretations is almost 100% (>95%) if the visual angles *θ_BC_*, *θ_CA_*, and *θ_AB_* are small (*θ*_max_ ≤ 14° in Figure 2A,B). We also found that the frequency of 2 possible interpretations decreases as the retinal image becomes larger. The number of possible interpretations is often 0 (>60%) if all the visual angles *θ_BC_*, *θ_CA_*, and *θ_AB_* are larger than 40° (*θ*_max_ ≥ 80° in Figure 2B). This number is rarely 0 (<1%) if all of the visual angles *θ_BC_*, *θ_CA_*, and *θ_AB_* are smaller than 20° (*θ*_max_ ≤ 20° in Figure 2A,B). The number of possible interpretations is rarely three or four for any value of *θ*_max_.

Note that the projection from the triangle to its retinal image is *perspective* but it can also be approximated well with an *orthographic* projection with uniform scaling when the visual angle of the triangle is small. The two possible interpretations of the small retinal image (*θ*_max_ ≤ 14° in Figure 2A,B) are analogous to the depth reversal ambiguity of an orthographic image of a 3D wire-frame object such as a Necker cube [29]. We confirmed that the orientations of the triangle in the two possible interpretations are approximately depth reversals of each other.

**Figure 3:**
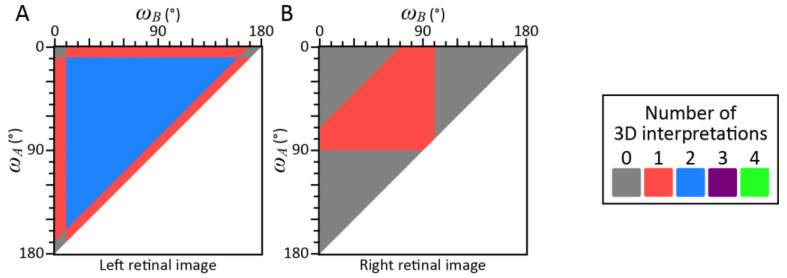
Results of the analyses of two retinal images of triangles where (*θ_BC_*, *θ_CA_*, *θ_AB_*) = (10°, 15°, 20°) in (**A**) and (90°, 100°, 110°) in (**B**). The ordinate and abscissa show two angles *ω_A_* and *ω_B_* of the triangle *ABC*. The colors indicate the number of possible interpretations of the triangle. White regions indicate where the shapes of the triangle would not be valid.

The number of possible interpretations of the triangle is almost always 1 or more than 1 if the image is sufficiently small (*θ*_max_ ≤ 20° in Figure 2A,B) but is often 0 if the image is sufficiently large (*θ*_max_ ≥ 80° in Figure 2B). These trends were examined by performing an additional analysis of the effect of small and large retinal images: (*θ_BC_*, *θ_CA_*, *θ_AB_*) = (9°, 10°, 11°) and (90°, 100°, 110°). The shapes of the triangles that could be projected to these images were computed by using the same program we used to solve the P3P problem. The results of these analyses are shown in Figure 3. Each point in these maps represents the shape of a triangle *ABC*. The abscissas and the ordinates show two angles *ω_A_* and *ω_B_* of the triangle *ABC*. The third angle *ω_C_* was computed as *ω_C_* = 180°—*ω_A_*—*ω_B_*. The colors of the point indicate the number of possible interpretations of the triangle with a specified shape (*ω_A_*, *ω_B_*, *ω_C_*). These trends were also observed in the individual retinal images. Almost any triangular shape (10° < *ω_A_*, *ω_B_*, *ω_C_* < 170°) can be projected to a small retinal image and there were two possible orientations for many of the individual shapes. With the large retinal images, less than half of the triangular shapes can be projected, so large retinal image restricts the shape of the triangle. This suggests that a large retinal image of a triangle can, to some extent, actually serve as a cue for the shape of the triangle.

### 2.2. Analyzing the Retinal Images of Triangles That Have Served as Visual Stimuli

We began by examining the shape of a triangle that was projected to a specified retinal image. We analyzed the retinal images of the triangles that had been used in (i) Beck & Gibson’s [30] Experiment 1, and in (ii) Watanabe’s [31] Condition 3, where they studied (i) the relationship between the perceived shape of the triangle and its orientation in a 3D scene and (ii) the distortion of a perceived 3D space by comparing the visual stimuli with their observers’ responses. Note that the Watanabe’s [31] paper provides important support for Indow’s [32] theory that perceived space is distorted and that this distortion is hyperbolic. We chose these studies because of the clarity of the authors’ descriptions of the visual stimuli they used in their experiments and the simplicity of their stimuli. In both of these experiments, the observers were shown the triangles in dark rooms and they responded (i) by constructing its shape and (ii) by adjusting the positions of its vertices as well as the positions of a few added points. The triangles were viewed monocularly in Beck & Gibson and binocularly in Watanabe. The shapes of the triangles that could be projected to retinal images were computed by using the program we used to solve the P3P problem.

**Figure 4:**
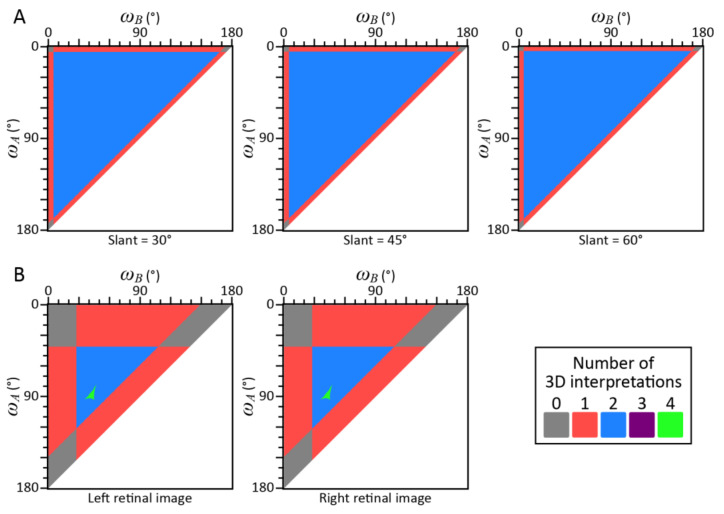
Results of the analyses of the retinal images of triangles: in (**A**) Beck & Gibson [30] and in (**B**) Watanabe [31]. The ordinate and abscissa show two angles *ω_A_* and *ω_B_* of the triangle *ABC*. The colors indicate the number of possible interpretations of the triangle. White regions indicate where the shapes of the triangle would not be valid. (**A**) The results of the analysis of the retinal images of the triangle that had 3 different orientations in Beck & Gibson [30]: (*θ_BC_*, *θ_CA_*, *θ_AB_*) = (5.538°, 5.538°, 6.573°), (4.928°, 4.928°, 6.638°), and (4.222°, 4.222°, 6.689°). (**B**) The results of the analysis of the retinal images in Condition 3 of Watanabe [30] with binocular viewing. His retinal images (*θ_BC_*, *θ_CA_*, *θ_AB_*) were (41.19°, 27.68°, 31.01°) for the left eye and (41.18°, 27.78°, 30.91°) for the right eye.

The results of these analyses are shown in Figure 4. Each point in these maps represents the shape of the triangle *ABC*. The abscissas and the ordinates show two angles *ω_A_* and *ω_B_* of the triangle *ABC*. The third angle *ω_C_* could be computed as *ω_C_* = 180°—*ω_A_*—*ω_B_*. Colors of the point indicate the number of possible interpretations of a triangle with a specified shape (*ω_A_*, *ω_B_*, *ω_C_*).

The three panels of Figure 4A show the number of possible interpretations for the retinal images of the triangle that had 3 different orientations in Beck & Gibson [29]: (*θ_BC_*, *θ_CA_*, *θ_AB_*) = (5.538°, 5.538°, 6.573°), (4.928°, 4.928°, 6.638°), and (4.222°, 4.222°, 6.689°). These images are small (*θ_BC_*, *θ_CA_*, *θ_AB_* < 10°), and they are consistent with almost any shape of the triangle. Moreover, note that usually there were only two possible interpretations for each shape and that the number of possible interpretations is never 3 or 4.

The left and right panels of Figure 4B show the number of possible interpretations of the left and right retinal images in Watanabe’s [31] Condition 3. The individual images cannot be projected from about 20% of the triangular shapes. The number of possible interpretations was often 1 or 2 and it was rarely 4. The number 3 was not observed. This ambiguity remained even when the test was done binocularly. Many triangular shapes can be projected to both of the retinal images.

The geometrical ambiguity of the visual stimuli shown in this analysis can explain the empirical results in Beck & Gibson [30] and in Watanabe [31]. In Beck & Gibson [30], as well as in Gottheil & Bitterman [33], Epstein, Bontrager, & Park [34], and Wallach & Moore [35], the observers were shown triangles with a variety of shapes and responded by trying to construct a similar triangular shape. A comparison of the physical shapes of the triangles with the perceived shapes, as represented by their constructions, served as the measure of shape constancy. All of these studies showed that shape constancy was very poor during monocular viewing and that it only improved somewhat during binocular viewing. This discrepancy between the physical and the perceived shapes of the triangles with binocular viewing was also observed by Watanabe [31]. Watanabe claimed that this occurred because the perceived space was distorted. However, his failure to achieve perfect, or near perfect, shape constancy can be explained more parsimoniously by the geometrical ambiguity of the visual stimuli used and not by defects in the visual systems of the observers.

## 3. General Discussion

This study examined how the shape and orientation of a triangle within a 3D scene can be recovered from its 2D retinal image. The orientation of the triangle can be arbitrary unless the normal to the plane of the triangle is perpendicular to any of the lines of the projection. Almost any triangular shape can be projected to the retinal image if the image is less than 20° (see Monte-Carlo simulation; *θ*_max_ ≤ 20° in Figure 2A,B, Figure 3A and Figure 4A). This ambiguity cannot be resolved even when the triangle is viewed binocularly (see our analyses of the images of the triangles that served as our visual stimuli; Figure 4B). When the retinal image is large, some shapes cannot be projected to the retinal image (see Monte-Carlo simulation; *θ*_max_ > 20° in Figure 2A,B and Figure 3B). This suggests that the retinal image of a triangle cannot serve as a cue for the shape of the triangle unless it is sufficiently large.

Now consider that if there are 4 feature points in a 3D scene, they usually form a volumetric polyhedron that has 4 vertices. This brings up the Perspective-4-Point (P4P) problem where one must recover the orientation of the polyhedron when the 3D shape and the 2D retinal image of the polyhedron are given [36]. It has been proven that there are 0 or up to 5 possible 3D interpretations of the polyhedron for a given shape, as well as for the size, and for the retinal image of the given polyhedron. Now, consider a case in which the 4 points are constrained to be coplanar to one another in the scene, and in which they form a planar polygon. Their orientation can be uniquely recovered from its monocular retinal image when the shape of the polygon is given [37,38]. A recovery is also usually possible from a stereo-pair of retinal images under the constraint that the shape is planar, but the shape of the polygon need not be given [39].

There needs to be 5, or more than 5, feature points in a 3D scene that project to a stereo-pair of retinal images if the 3D scene is going to be recovered from the stereo-pair [40,41]. A triangle has only 3 vertices so these are not sufficient to execute a recovery. The recovery becomes possible if there are 2, or more than 2, additional feature points in the scene [42]. A shortage of visual information on the retinas can also be compensated by using oculomotor information about the orientations of the eyes relative to the head. Note that the orientations of the eyes can be estimated by using the efference signal produced by the oculomotor control system.

Other “simple” 3D visual stimuli, such as an ellipse [43], and points on the sagittal plane that bisects the interocular axis perpendicularly [39], as well as points on a plane coplanar with the eyes [44], present analogous problems. Note that many psychophysical studies have shown that perception is not veridical and that percepts are distorted when such simple visual stimuli are used (e.g., [32]). However, note that such distortions could be attributed to defects in another mechanism that is being used to compensate for the shortage of visual information inherent in the too simple visual stimuli, such as the oculomotor efference signal. Note that other studies have shown that our perception of 3D scenes and the shapes of 3D objects is veridical in everyday life [4,45].

In our everyday life, 3D scenes “out there” are complex and it is this complexity that plays the critical role in perceiving them veridically. There are usually many feature points in a natural 3D scene and these points are essential for the binocular recovery of 3D [45,46] particularly when they become more widely distributed [44,47,48] and if they have sufficient density [49]. These feature points are inherent in the 3D objects present in the scene. Note that: (i) the shapes and positions of these objects often satisfy a number of a priori constraints, and (ii) the visual system can make use of these a priori constraints to recover a 3D scene from its 2D representation on the retina [4,5,6,7] (see Reference [50] for example). In light of these facts, it is questionable that many, if any, inferences about the visual system can be generalized to the veridical perception observed in our everyday life from the non-veridical perceptions that have been observed in many psychophysical studies that used very simple visual stimuli, such as triangles, ellipses, and planes that intersect the interocular axis perpendicularly, or are coplanar with the eyes. The visual information required to recover 3D scenes is absent when these simple visual stimuli are used. Human performance observed under such deprived conditions cannot be generalized to performance under natural viewing conditions.

These simple visual stimuli were selected and used because they provided a convenient way to eliminate artifacts from the visual stimuli and to facilitate control of the experiment. The goal of our study was to *explain why* using a triangle introduces ambiguity that does not exist when more complex stimuli are used. The results of our simulation experiments show clearly that we achieved our goal. This encourages us to conclude by emphasizing that understanding the theoretical properties of one’s visual stimuli is critical for designing experiments concerned with shape and depth and for interpreting the results obtained.

## Data Availability

The C++ code used in this study is openly available in GitHub [51].

## Appendix A

The Perspective-3-Point (P3P) problem is concerned with recovering the orientation of a triangle *ABC* from its retinal image when the 3D shape of *ABC* and its 2D retinal image are given [19,20,21,22,23,24,25,26,27,28]. There are 0 or up to 4 possible 3D interpretations of the triangle. An algorithm developed by Fischler & Bolles [21] for solving the P3P problem was implemented in a C++ program and used for the simulations in this study. This algorithm recovers the distance of the vertices *A*, *B*, and *C* of the triangle from a center of projection *E*. Let the distance of the vertices *A*, *B*, and *C* be *l_A_*, *l_B_*, and *l_C_*. The output of this algorithm is vectors with 3 values. These vectors represent individual 3D interpretations of the triangle and the 3 values of the vectors represent *l_A_*, *l_B_*, and *l_C_*.

There are two important properties of the algorithm that were used in our implementation. First, the algorithm needs to solve a quartic equation for recovering *l_A_*, *l_B_*, and *l_C_*. The roots of this quartic equation were computed by using Ferrari’s method [52]. Ferrari’s method was implemented in a C++ function based on [53].

Second, the algorithm recovers the distance *l_A_*, *l_B_*, and *l_C_* of the vertices *A*, *B*, and *C* in an unbalanced manner. The distance to 2 of the 3 vertices is recovered first and the distance to the last vertex is recovered on the basis of the recovered distance to the first 2 vertices. In a few rare cases, the results of the recovery changes depending on which vertex was recovered last because of rounding and discretization errors. This problem was addressed in our implementation by recovering *l_A_*, *l_B_*, and *l_C_* with 3 different orders in which the last recovered distance was different from one another. Note that a result of the recovery was vectors of the recovered *l_A_*, *l_B_*, and *l_C_*. Sets of the recovered vectors from the 3 different orders were combined and duplications of the vectors were eliminated. The vectors were also verified by recovering the 3D shape of *ABC* from its 2D retinal image and from each recovered vector. The vector was eliminated whenever the recovered shape was substantially different from the given shape of *ABC*. The C++ code for this implementation was uploaded to GitHub [51].

Our implementation of the algorithm was tested in a simulation experiment. In each trial of this experiment, a triangle was randomly generated in a 3D scene and its retinal image was computed. The 3D XYZ Cartesian coordinate system was set in the 3D scene to place the origin at the center of projection *E*. The vertices *A*, *B*, and *C* were placed in the scene so that their Z-coordinates were between 1 and 100. Angles between the *Z*-axis and lines of projection to *A*, *B*, and *C* were less than *ε*, where *ε* is 45° in one condition and is 85° in the other condition, and 10^6^ scenes were randomly generated for each condition.

In each trial, the algorithm recovered the depth *l_A_*, *l_B_*, and *l_C_* of the triangle’s vertices *A*, *B*, and *C* from the retinal image of the simulated scene and the simulated shape of the triangle. The recovered depth was compared with the simulated depth of the vertices as follows:

(A1) $$\delta ={\left(\frac{l_{A}}{\left\|l\right\|}-\frac{l{'}_{A}}{\left\|l'\right\|}\right)}^{2}+{\left(\frac{l_{B}}{\left\|l\right\|}-\frac{l{'}_{B}}{\left\|l'\right\|}\right)}^{2}+{\left(\frac{l_{C}}{\left\|l\right\|}-\frac{l{'}_{C}}{\left\|l'\right\|}\right)}^{2}\;\left\{\begin{array}{c} \|l\|=\sqrt{l_{A}{}^{2}+l_{B}{}^{2}+l_{C}{}^{2}}\\ \|l'\|=\sqrt{l{'}_{A}{}^{2}+l{'}_{B}{}^{2}+l{'}_{C}{}^{2}} \end{array}\right.$$

where *l_A_*, *l_B_*, and *l_C_* are the simulated depth of the vertices *A*, *B*, and *C* and *l*′*_A_*, *l*′*_B_*, and *l*′*_C_* are their recovered depth. The recovered depth was considered to be veridical if the difference δ between the simulated and recovered depth was less than 0.01.

The depth of the same 10^6^ scenes were also recovered by using 3 other algorithms that are used to solve the P3P problem, specifically, Gao, Hou, Tang, & Chang [22]; Ke & Roumeliotis [25]; and Banno [27]. The algorithms of Gao, Hou, Tang, & Chang [22] and Ke & Roumeliotis [25] were implemented as a function in the OpenCV library (ver. 4.2.0, [54,55]). The algorithm of Banno [27] was implemented by Banno himself [27]. The performance of all of these 3 implementations was compared with our implementation.

The performance of these implementations is shown in Table A1. Their performance was evaluated by examining the probability of failing to recover the depth of the simulated scenes and the time required to recover the scenes. Note that the retinal images were projections of the simulated scenes and the simulated shapes of the triangles were given for the recovery. Hence, possible interpretations of the retinal images should have included the simulated scenes. Our implementation always recovered the simulated 3D scene from its retinal image. The other 3 algorithms failed to recover some of the simulated scenes. Our implementation’s processing speed was also the highest.

**Table A1:** Performance of the 4 implementations of the P3P algorithms used to recover a 3D scene with a randomly-generated triangle. The implementations were evaluated on the basis of their recovery of 10^6^ scenes. The probability of the failure to recover the simulated scene and the time required for processing the recovery of the 10^6^ scenes were measured.

Algorithms	*ε*	% Failed Trial	Processing Time
Minkov & Sawada	45°	0%	2.7 s
	85°	0%	2.6 s
Gao, Hou, Tang, & Chang [22]	45°	0.034%	20 s
	85°	0.025%	18 s
Ke & Roumeliotis [25]	45°	21%	25 s
	85°	25%	26 s
Banno [27]	45°	29%	8.7 s
	85°	22%	7.8 s

The performance of our implementation was also tested with different values of the threshold for the depth difference δ. The implementation continued to perform perfectly when the threshold was set to 10^−6^ or larger and only a few errors (0.0041%) were made when the threshold was set to 10^−7^. Our implantation of the algorithm used for solving the P3P problem was highly reliable.
